# Supplementary material for: A contraction approach to dynamic optimization problems
Source: PLoS One. 2021 Nov 22;16(11):e0260257. doi: 10.1371/journal.pone.0260257 (PMC8608347; doi:10.1371/journal.pone.0260257)
Supplement: S1 File — (PDF) [file pone.0260257.s001.pdf]

Pseudo-code for example 1 in
A contraction approach to dynamic optimization problems
by
L.K. Sandal, S.F. Kvamsdal, J. Maroto, M. Morán

Example 1: Periodic price in a resource model (section 3.1)

The central part, the optimization, consists of policy and value iterations. The policy iteration effectuates the maximum operator in equation (5) for a given value function, calculating the optimal policy. The value iteration presumes a given policy as optimal and updates the value function. The same structure is used for the contraction solution of example 2 (section 3.2), but is then modified to a two-dimensional state space.

Setup, load model

- Define state space grid
- Define parameter values
- Define growth operator ('F') and return functions ('Pi_k')
- Define first-order-conditions (FOC) for inner solutions as functions of the value function gradients

Optimization

- Initiate the value functions ('V_k = 0')
- Define convergence condition
- While-loop [while condition not met]
    - Policy iteration
        - Calculate gradients of value functions
        - Determine optimal policy (inner or boundary)
            - If FOC not intersects zero, choose boundary solution
            - If FOC intersects zero, choose inner solution
    - Value iteration
        - For-loop, iterate on equation (5) for given policy
    - Calculate condition (convergence criteria) test
