# Supplementary material for: A contraction approach to dynamic optimization problems
Source: PLoS One. 2021 Nov 22;16(11):e0260257. doi: 10.1371/journal.pone.0260257 (PMC8608347; doi:10.1371/journal.pone.0260257)
Supplement: S2 File — (PDF) [file pone.0260257.s002.pdf]

Code for example 2 (section 3.2) in
A contraction approach to dynamic optimization problems
by
LK Sandal, SF Kvamsdal, M Maroto, M Morán

Code for establishing the exact solution. Equation references in the comments refer to equation numbers in the article.

```
> restart;
```

Define parameter values

```
> a := 0.5; b := 0.5; c01 := 0.4; c02 := 0.3; c11 := 0.2; c12 := 0.1; beta1 := 0.95; beta2 := 0.8;
```

$$a := 0.5$$
$$b := 0.5$$
$$c01 := 0.4$$
$$c02 := 0.3$$
$$c11 := 0.2$$
$$c12 := 0.1$$
$$\beta1 := 0.95$$
$$\beta2 := 0.8 \tag{1}$$

Define price function

```
> p := 1 - u1 - u2;
```

$$p := 1 - u1 - u2 \tag{2}$$

Define cost functions

```
> c1 := c01 - c11·x;
```

$$c1 := -0.2\,x + 0.4 \tag{3}$$

```
> c2 := c02 - c12·y;
```

$$c2 := -0.1\,y + 0.3 \tag{4}$$

Define return functions

```
> Pi1 := (p - c1)·u1;
  Pi2 := (p - c2)·u2;
```

$$\pi1 := (0.6 - u1 - u2 + 0.2\,x)\,u1$$
$$\pi2 := (0.7 - u1 - u2 + 0.1\,y)\,u2 \tag{5}$$

Define state transition, equation (16)

```
> X := a + b·x - u1;
  Y := a + b·y - u2;
```

$$X := -u1 + 0.5\,x + 0.5$$
$$Y := -u2 + 0.5\,y + 0.5 \tag{6}$$

Define expressions for value functions, equation (18)

```
> V1 := F1 + A1·x + B1·y + C1·x^2 + D1·x·y + E1·y^2;
  V2 := F2 + A2·x + B2·y + C2·x^2 + D2·x·y + E2·y^2;
```

$$V1 := C1\,x^2 + D1\,x\,y + E1\,y^2 + A1\,x + B1\,y + F1$$
$$V2 := C2\,x^2 + D2\,x\,y + E2\,y^2 + A2\,x + B2\,y + F2 \tag{7}$$

Differentiate value functions with respect to the state variables

```
> DV1 := diff(V1, x);
  DV2 := diff(V2, y);
```

$$DV1 := 2\,C1\,x + D1\,y + A1$$

$$DV2 := D2\,x + 2\,E2\,y + B2 \quad \textbf{(8)}$$

Substitue in state transition, equation (16), into derivatives of the value functions

```
> DVV1 := subs(x = X, y = Y, DV1);
  DVV2 := subs(x = X, y = Y, DV2);
```

$$DVV1 := 2\,C1\,(-u1 + 0.5\,x + 0.5) + D1\,(-u2 + 0.5\,y + 0.5) + A1$$

$$DVV2 := D2\,(-u1 + 0.5\,x + 0.5) + 2\,E2\,(-u2 + 0.5\,y + 0.5) + B2 \quad \textbf{(9)}$$

Define first-order-conditions, derivatives of the argument of the maximum operator in equation (17)

```
> FOC1 := diff(Pi1, u1) - beta1·DVV1;
  FOC2 := diff(Pi2, u2) - beta2·DVV2;
```

$$FOC1 := -2\,u1 + 0.6 - u2 + 0.2\,x - 1.90\,C1\,(-u1 + 0.5\,x + 0.5) - 0.95\,D1\,(-u2 + 0.5\,y + 0.5) - 0.95\,A1$$

$$FOC2 := -2\,u2 + 0.7 - u1 + 0.1\,y - 0.8\,D2\,(-u1 + 0.5\,x + 0.5) - 1.6\,E2\,(-u2 + 0.5\,y + 0.5) - 0.8\,B2 \quad \textbf{(10)}$$

Solve first-order-conditions to obtain algebraic expressions for the optimal decision variables

```
> FOCsol := solve({FOC1 = 0, FOC2 = 0}, {u1, u2});
```

$$FOCsol := \{u1 = (0.5000000000\,(304.\,C1\,E2\,x - 76.\,D1\,D2\,x + 304.\,A1\,E2 - 152.\,B2\,D1 + 304.\,C1\,E2 - 380.\,C1\,x - 76.\,D1\,D2 - 171.\,D1\,y + 80.\,D2\,x - 64.\,E2\,x + 160.\,E2\,y - 380.\,A1 + 160.\,B2 - 380.\,C1 - 57.\,D1 + 80.\,D2 - 32.\,E2 + 80.\,x - 20.\,y + 100.))\,/\,(304.\,C1\,E2 - 76.\,D1\,D2 - 380.\,C1 + 95.\,D1 + 80.\,D2 - 320.\,E2 + 300.),\ u2 = -(0.5000000000\,(-304.\,C1\,E2\,y + 76.\,D1\,D2\,y + 152.\,A1\,D2 - 304.\,B2\,C1 - 304.\,C1\,E2 - 190.\,C1\,x + 38.\,C1\,y + 76.\,D1\,D2 - 95.\,D1\,y + 128.\,D2\,x + 320.\,E2\,y - 190.\,A1 + 320.\,B2 + 76.\,C1 - 95.\,D1 + 64.\,D2 + 320.\,E2 + 40.\,x - 40.\,y - 160.))\,/\,(304.\,C1\,E2 - 76.\,D1\,D2 - 380.\,C1 + 95.\,D1 + 80.\,D2 - 320.\,E2 + 300.)\} \quad \textbf{(11)}$$

Assign algebraic expressions to the decision variable names

```
> assign(FOCsol) :
```

Define equations governing the valuefunctions for given optimal decision variables

```
> ME1 := sort(Pi1 + beta1·subs(x = X, y = Y, V1) - V1, [x, y]) :
  ME2 := sort(Pi2 + beta2·subs(x = X, y = Y, V2) - V2, [x, y]) :
```

Establish expressions for coefficients in equation (18) from the governing equations given optimal decision variables

```
> L1 := subs(x = 0, y = 0, diff(ME1, x)) :
> L2 := subs(x = 0, y = 0, diff(ME1, y)) :
> L3 := subs(x = 0, y = 0, diff(ME1, x, y)) :
> L4 := subs(x = 0, y = 0, diff(ME1, x, x)) :
> L5 := subs(x = 0, y = 0, diff(ME1, y, y)) :
> L6 := subs(x = 0, y = 0, diff(ME2, x)) :
  L7 := subs(x = 0, y = 0, diff(ME2, y)) :
  L8 := subs(x = 0, y = 0, diff(ME2, x, y)) :
  L9 := subs(x = 0, y = 0, diff(ME2, x, x)) :
  L10 := subs(x = 0, y = 0, diff(ME2, y, y)) :
  L11 := subs(x = 0, y = 0, ME1) :
  L12 := subs(x = 0, y = 0, ME2) :
```

Numerical solutions for coefficients

> $test := fsolve(\{L1, L2, L3, L4, L5, L6, L7, L8, L9, L10, L11, L12\}, \{A1, A2, B1, B2, C1, C2, D1, D2, E1, E2, F1, F2\});$

$$test := \{A1 = 0.07253707576, A2 = -0.04642775724, B1 = -0.01837518221, B2 = 0.05589251338, C1 = 0.01938142440, C2 = 0.004148927536, D1 = -0.009524565372, D2 = -0.009012601496, E1 = 0.001170692788, E2 = 0.004960927749, F1 = 1.010140851, F2 = 0.3895827515\} \quad \textbf{(12)}$$

Numeric solutions for decision and value functions

> $U1 := subs(test, u1);$

$$U1 := 0.1237184940\, x - 0.03036429332\, y + 0.1276409664 \quad \textbf{(13)}$$

> $U2 := subs(test, u2);$

$$U2 := -0.06074381345\, x + 0.06355949130\, y + 0.2642291673 \quad \textbf{(14)}$$

> $W1 := subs(test, V1);$

$$W1 := 0.01938142440\, x^2 - 0.009524565372\, x\, y + 0.001170692788\, y^2 + 0.07253707576\, x - 0.01837518221\, y + 1.010140851 \quad \textbf{(15)}$$

>

> $W2 := subs(test, V2);$

$$W2 := 0.004148927536\, x^2 - 0.009012601496\, x\, y + 0.004960927749\, y^2 - 0.04642775724\, x + 0.05589251338\, y + 0.3895827515 \quad \textbf{(16)}$$
