# Supplementary material for: A contraction approach to dynamic optimization problems
Source: PLoS One. 2021 Nov 22;16(11):e0260257. doi: 10.1371/journal.pone.0260257 (PMC8608347; doi:10.1371/journal.pone.0260257)
Supplement: S1 Appendix — (DOCX) [file pone.0260257.s003.docx]

# Appendix

To formulate the periodic problem as an extension of equation (1) and see that this formulation leads to equation set (5), let, for a given decision vector $u=(u_{1},\ldots,u_{N})$:

|  | ${\beta\tilde{\Pi}}_{1}\left( x,u \right)=\beta_{1}\Pi_{1}\left( y_{1},u_{1} \right)+\beta_{1}\beta_{2}\Pi_{2}\left( y_{2},u_{2} \right)+\ldots+\beta_{1}\beta_{2}\cdots\beta_{N}\Pi_{N}\left( y_{N},u_{N} \right)$ $=\sum_{j=1}^{N} \tilde{\beta}_{j}\Pi_{j}\left( y_{j},u_{j} \right)$ | (A1) |
| --- | --- | --- |

In equation (A1), we write $\tilde{\beta}_{j}=\beta_{1}\cdots\beta_{j}=\prod_{i=1}^{j} \beta_{i}$, $y_{1}=x$, and $y_{k+1}=F_{k}(y_{k},u_{k})$. As in the main text, the discount factor for $N$ intervals is $\beta=\prod_{i=1}^{N} \beta_{i}=\tilde{\beta}_{N}$. If we consider equation (1) with $\tilde{\Pi}_{1}$ as the objective function, we have what we call $V_{1}(x)$:

|  | $V_{1}\left( x \right)=\max_{\left\{ u_{kj} \right\}} \sum_{k=0}^{\infty} \beta^{k+1}\tilde{\Pi}_{1}\left( x_{k},u_{k} \right)=\max_{\left\{ u_{kj} \right\}} \sum_{k=0}^{\infty} \beta^{k}\sum_{j=1}^{N} \tilde{\beta}_{j}\Pi_{j}\left( y_{j},u_{kj} \right)$ | (A2) |
| --- | --- | --- |

Equations (A1) and (A2) presume that interval 1 is the initial interval in the decision problem. If the initial interval is interval $s$, $1<s\leq N$, the relevant value function, denoted $V_{s}(x)$, can be expressed as follows:

|  | $V_{s}\left( x \right)=\max_{\left\{ u_{kj} \right\}} \sum_{k=0}^{\infty} \beta^{k}\sum_{j=s}^{N+s-1} \frac{\tilde{\beta}_{j}}{\tilde{\beta}_{s-1}}\Pi_{j}\left( y_{j},u_{kj} \right)=\max_{\left\{ u_{kj} \right\}} \sum_{k=0}^{\infty} \beta^{k+1}\tilde{\Pi}_{s}(x_{k},u_{k})$ | (A3) |
| --- | --- | --- |

Given the periodic features of $\Pi_{k}$ and $F_{k}$, and thus $\tilde{\Pi}_{s}$, we see by inspection that $V_{s+N}\left( x \right)=V_{s}(x)$. In this sense, $V_{s}(x)$ is periodic; the decision problem one faces every $N$ intervals has the same structure. Furthermore, if $V_{s+1}(y)$ is known for all feasible $y=F_{s}(x,u)$, we have:

|  | $V_{s}\left( x \right)=\max_{\{u\}} \left\{ \beta_{s}\Pi_{s}\left( x,u \right)+\beta_{s}V_{s+1}(y) \right\}$ | (A4) |
| --- | --- | --- |

Equation (A4) follows from the principle of optimality.
