# Supplementary material for: A contraction approach to dynamic optimization problems
Source: PLoS One. 2021 Nov 22;16(11):e0260257. doi: 10.1371/journal.pone.0260257 (PMC8608347; doi:10.1371/journal.pone.0260257)
Supplement: Attachment — Submitted filename: PONE_RepliesToReviewers.pdf [file pone.0260257.s004.pdf]

Response to Reviewers

Manuscript PONE-D-21-01335

A contraction approach to dynamic optimization problems

First, let us express our gratitude towards the reviewers who have devoted time and effort to review our work. Their comments and suggestions have helped us improve our manuscript considerably. Below, we account for our revisions in context of the reviewer comments. Our replies are emphasized in *italics*. Comments of a technical nature, such as missing equation numbers and so on, are taken care of but omitted from the list below.

Reviewer #1: Abstract-

Add the evaluation and results achieved

> *We have revised the abstract. Our main finding, that what we refer to as periodic problems are part of a more general class of solvable contraction problems, were already mentioned in the abstract. We have highlighted this further. In the revised abstract, we refer some of the results from the examples as well.*

Introduction-

some references are needed when term periodicity is discussed.

> *We have added a reference to Kvamsdal et al. (2020), who also consider periodic problems, although in a narrower sense than what we do in the present manuscript. We would also like to point out that periodicity is considered a basic concept in dynamic systems. The third paragraph, beginning on line 54 in the revised manuscript, discusses the term and what we presently mean by it.*

Try to avoid using 'see' a lot to refer to existing works.

> *We agree and have revised the manuscript accordingly.*

Line108: "...of problems of type (1)." <-- Unclear what type(1) means. Is it the equation?

> *Yes, we mean the equation. We have revised the manuscript for clarity on this point.*

Line117" "..classical outset yielding (1) is then.." again please mention Equation (1) so if it's the equation

*Yes, again we mean the equation. We have revised the manuscript accordingly.*

Line126: Fig1- the variable k does not appear in the figure. Why?

*The variable $k$ is the interval index. We agree that Figure 1 was unclear and have revised the figure to clarify the role of $k$.*

Also I suggest to have a clearer section to explain the steps taken in this research. It is a bit unclear generally.

*We agree that the manuscript would benefit from an overview of how it is organized. We have added, to the end of the introduction section, a paragraph that outlines the various steps in the manuscript. The paragraph starts on line 99 in the revised manuscript.*

Line233: The Section name can be improved. Separate the sections for the two examples or create a subsection, with clearer name of the examples being used.

*We have revised the section name and added subsections for each of the examples, in accordance with the suggestion from the reviewer.*

Clearer explanation on the problem being used as example is needed.

*We have added some further explanation of both examples. For the first example, we have also moved discussion of related work from the end of section 2 to the example subsection (3.1).*

Line287: For second example, can be put in different section. Further explanation of the problem is needed, before going into the technicalities.

*We agree that the second example required further explanation. The example is now more carefully explained before getting to the technicalities. We have also revised the example throughout.*

Equation 15 not explained well.

*Earlier equation (15), now equation (17), is now explained better. In particular, the potentially confusing factor, the different role of the index $k$ from the previous example, is explained in a separate paragraph that starts on line 337 in the revised manuscript.*

Line306: How are these set of parameters derived? Any reference on the values used here?

*The set of parameters are simply chosen for numerical illustration. The manuscript has been revised to make this clear.*

Equation 16 and 17 that follow after the parameter, are not well explained.

*We have added brief explanations of the equations that now are numbered (19) and (20). Further, the supply functions in equation (20) are illustrated in the added Figure 3.*

Line311: The values shown in Equation 18 do not indicate that (18) is an equation.

*We agree that this is not an equation and have revised the manuscript accordingly.*

Outcome from Second Example is not very clear. Where is the outcome/results actually?

*In response to several of the comments from the reviewer, the second example is now better explained, and we hope the outcomes are clearer. We have illustrated the optimal decision rules in the new Figure 3. Let us hasten to add that the main purpose of the example was to show that the iterative solution converged to the exact solution. The example has been further revised on this point.*

Line314: "We discovered the above results"... avoid these kind of phrase. Please point exactly what is referred or at which section

*We had the main theorem in mind and have revised the manuscript accordingly.*

Lines 375-377: "As our suggestions for applications to dynamic games and regime shifts demonstrate, our result applies further to problems with a genuinely different structure than the classical Bellman problem" <-- Discuss this further. Why this is seen to be important discovery and how this will make you move forward with future related research?

*We found this suggestion a bit confusing. The paragraph was meant to briefly summarize the discussion of the various applications that we discuss earlier in the section. What we have done in our revision, however, is to add a brief paragraph prior to this final paragraph. In the added paragraph, we discuss work in bioeconomics that could be directly influenced by our contribution.*

OVERALL: The writing flow needs to be majorly improved. The structure (with sections) needs to be improved as well. It seems like the focus is put more on showing the formulas but not so much on the discussion and the steps taken on how these experiments are done.

*We have revised the manuscript to improve the writing flow. Several of the revisions resulting from other, more specific comments from both reviewers have also contributed to an improved flow. Finally, we have had the manuscript processed by a professional copyeditor.*

Line401: More References needs to be added ~ at least 4-5 more recent papers

*Four references from the last decade has been added at various places throughout the manuscript.*

Reviewer #2: The authors present an extension of the classical Bellmann optimization algorithm to periodic control problems. The work is presented in a clear and technically sound manner. As my only minor comment, I suggest that the authors give a few more details how periodicity in optimal control problems is handled by now (they write "... is often treated in

some ad-hoc manner" in the introduction). Also one or two references to such "ad-hoc" treatments would be appropriate in the first paragraph of the introduction.

> *While we never meant it that way, we realize that the term 'ad-hoc' could be understood as criticism. The point being that before a generic treatment like ours had been proposed, any approach would necessarily be ad-hoc in the literal sense of being for special purpose. We have thus avoided this term. Further, we have revised the first paragraph of the introduction and provide an example of practice that could be questioned. We also refer to some articles that illustrate, by mean of examples, that proper modeling of periodicity in optimal control problems matter. Notably, we have kept this discussion brief at this point in the manuscript. The examples are discussed somewhat further in the examples section. In addition, a brief paragraph prior to the final paragraph has been added where we discuss work in bioeconomics that could be directly influenced by our contribution.*
